# Supplementary material for: Plagued by a cryptic clock: insight and issues from the global phylogeny of Yersinia pestis
Source: Commun Biol. 2023 Jan 19;6:23. doi: 10.1038/s42003-022-04394-6 (PMC9852431; doi:10.1038/s42003-022-04394-6)

Plagued by a cryptic clock

Insight and issues from the global phylogeny of *Yersinia pestis*

Katherine Eaton[1,2], Leo Featherstone[3], Sebastian Duchene[3], Ann G. Carmichael[4], Nükhet Varlık[5], G. Brian Golding[6], Edward C. Holmes[7], Hendrik N. Poinar*[1,2,8,9,10]

SUPPLEMENTARY FIGURES:

Figure S1: Root to tip regression for clades of *Yersnia pestis as defined by the global phylogeny*

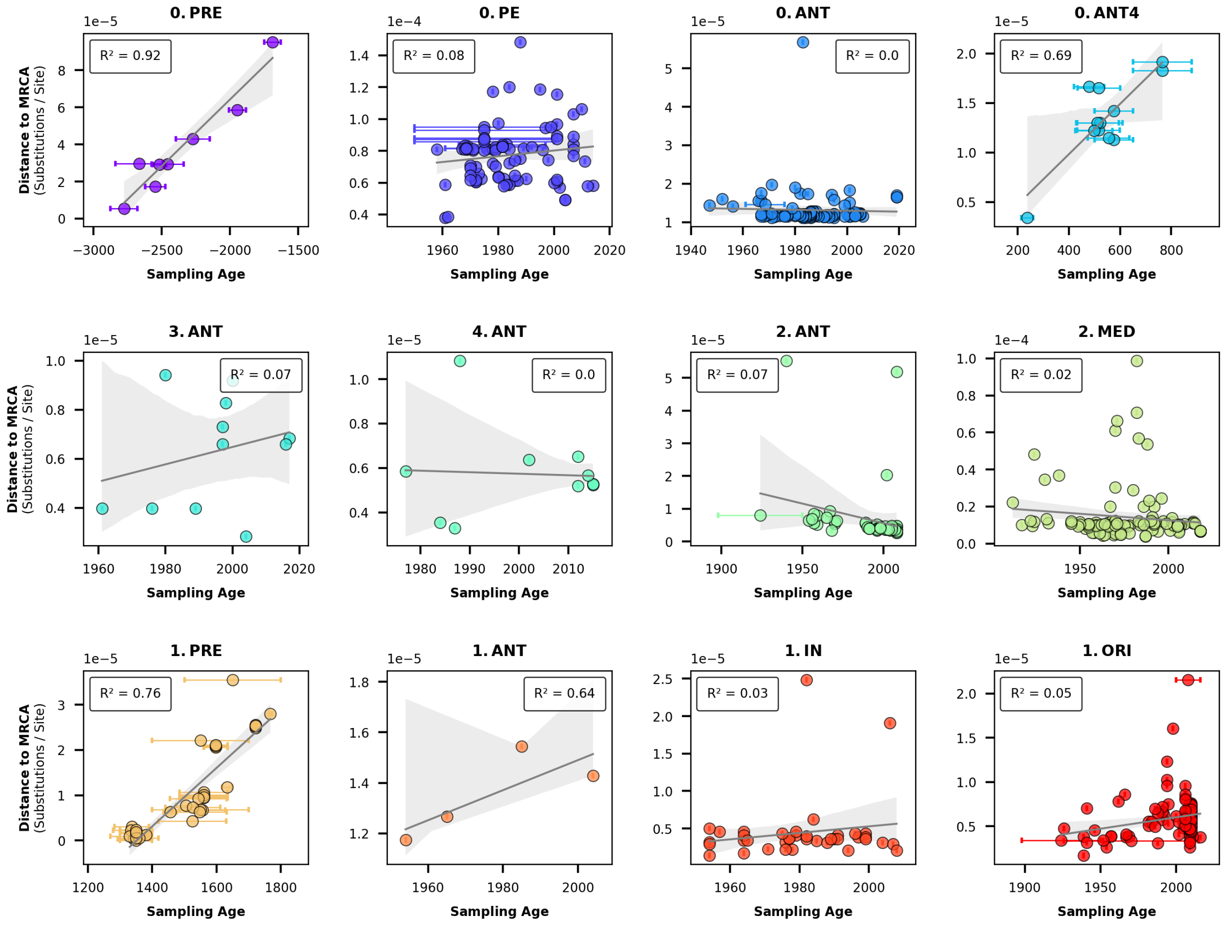

Figure S2: Nodal density vs root nodes for each clade of *Yersinia pestis*

A. No Temporal Signal

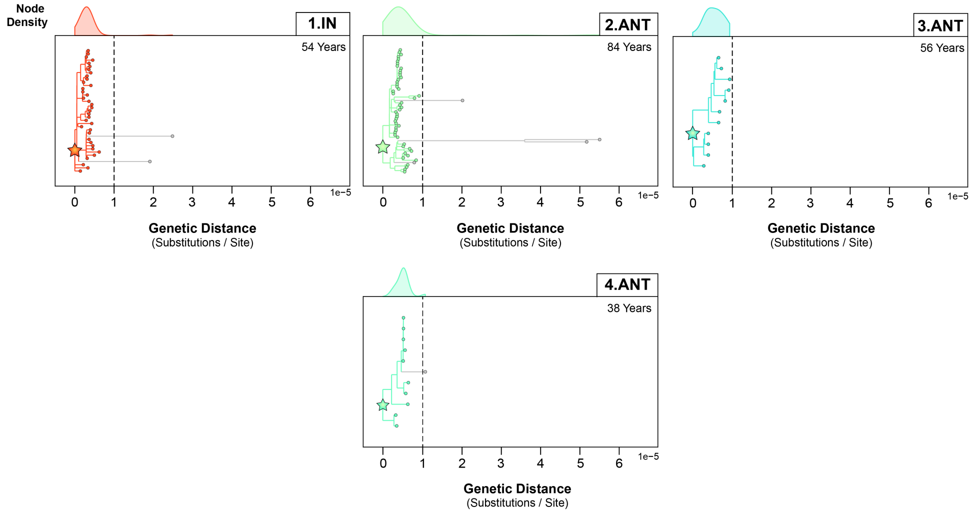

B. Insufficient Internal Calibrations

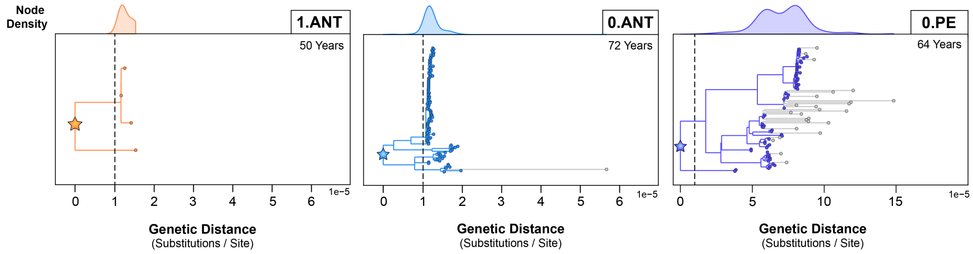

C. Temporal Signal With Informative Dates

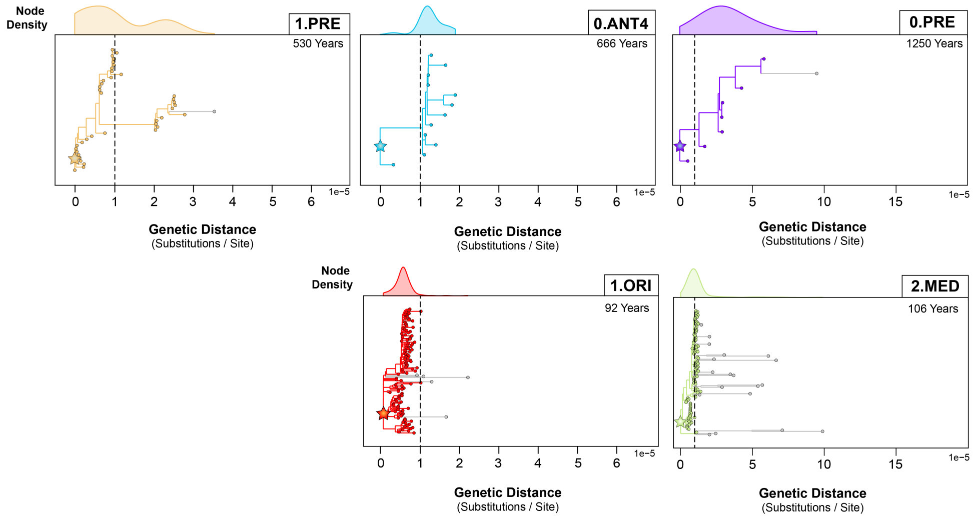

Figure S3: Branch support across MCC Tree of Second Pandemic of Plague

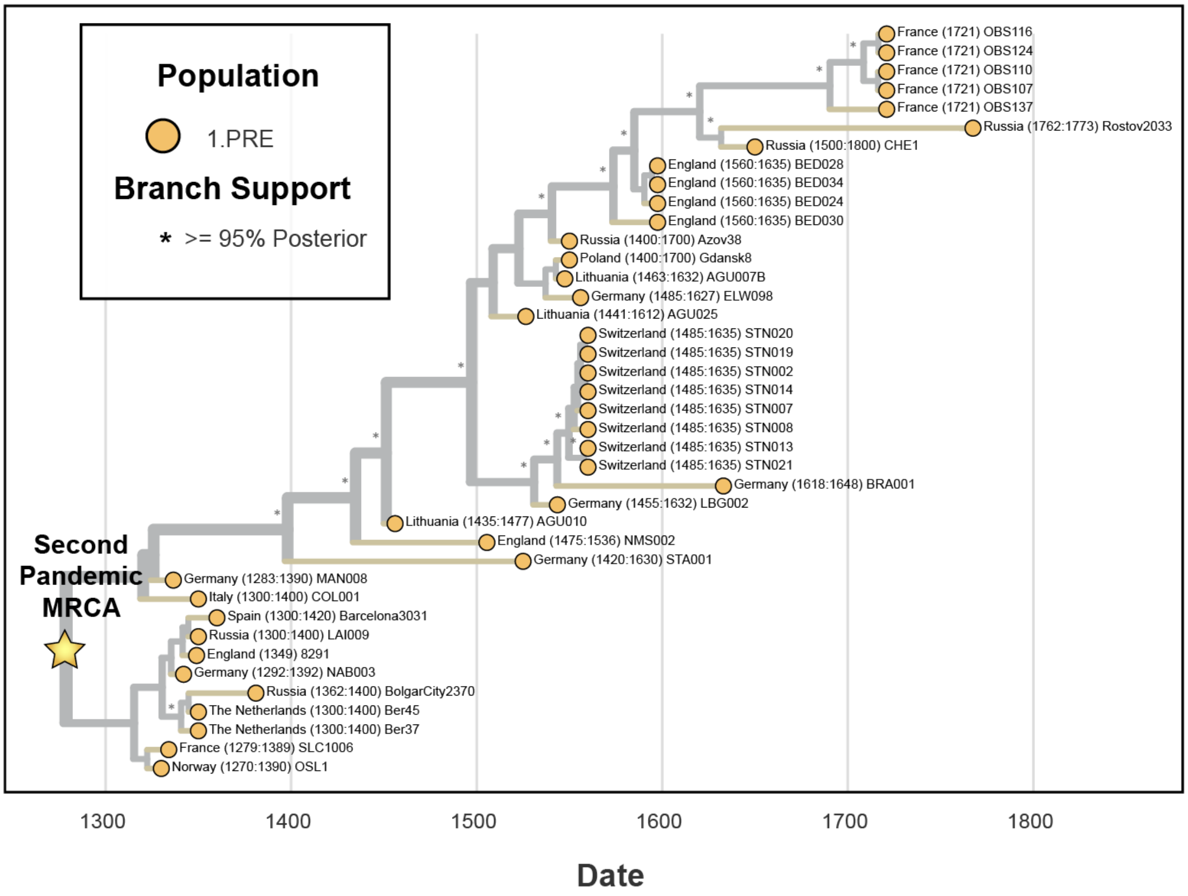

Figure S4: Percent of Samples Collected from the Same Location as their Closest Genetic Relative

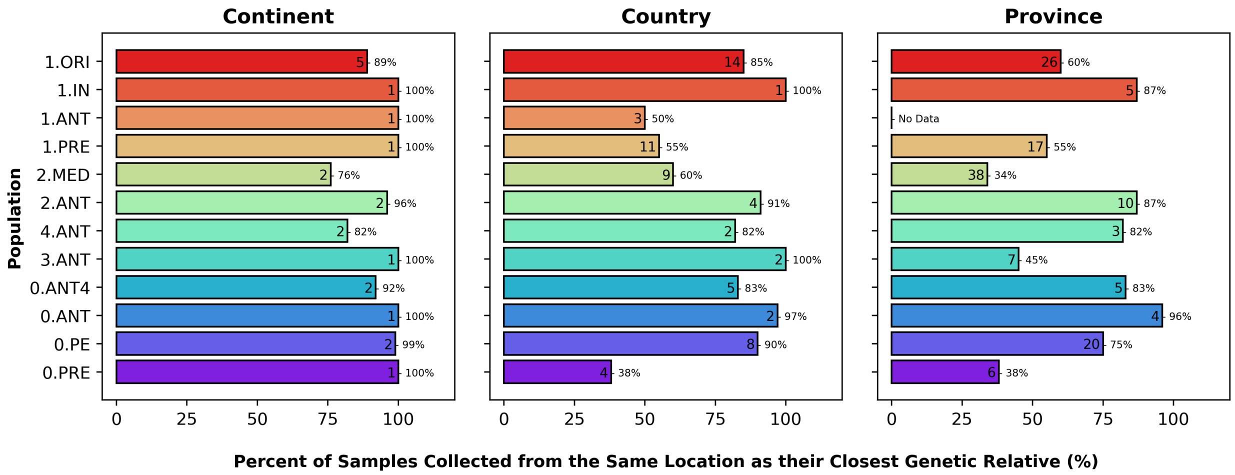

Figure S5: Confidence in Estimating the ancestral location (at varying geographic levels) for the Third Pandemic

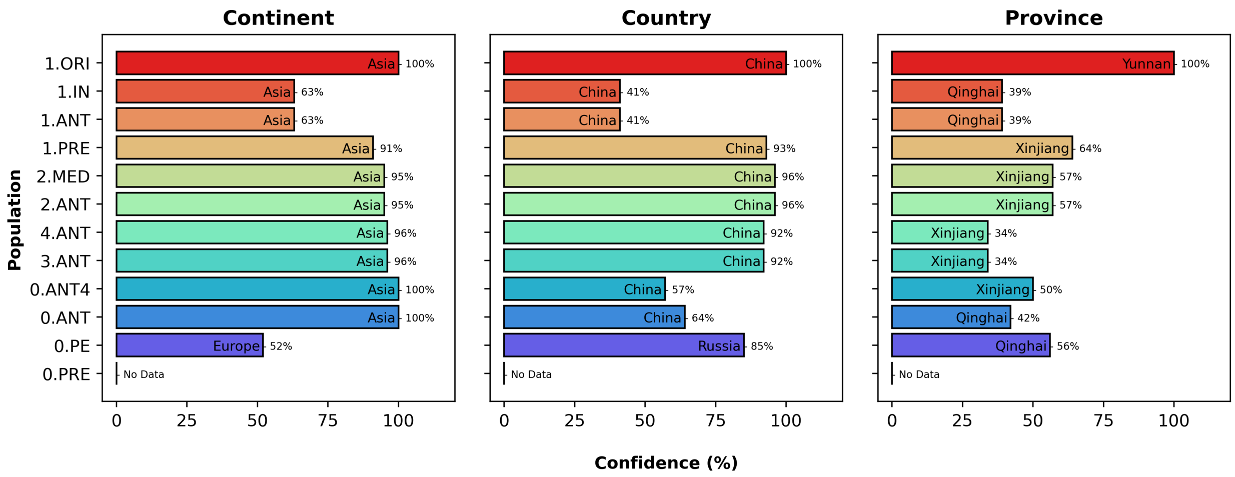

Figure S6: Maximum Likelihood Phylogeny *of Yersinia pestis* of Third Pandemic and the geographic location of phylogenetically closest basal Lineages

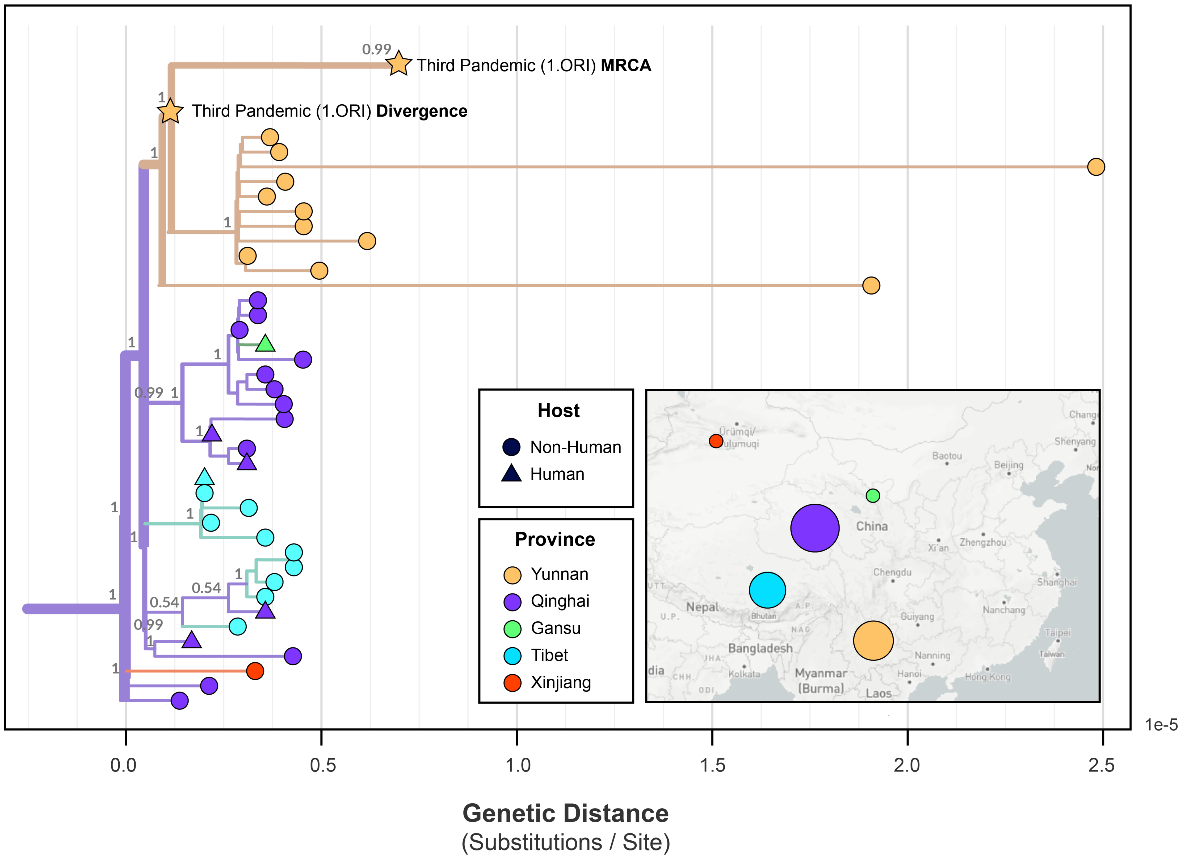

Figure S7: Confidence in Estimating the ancestral location (at varying geographic levels) for all other populations of *Y. pestis*

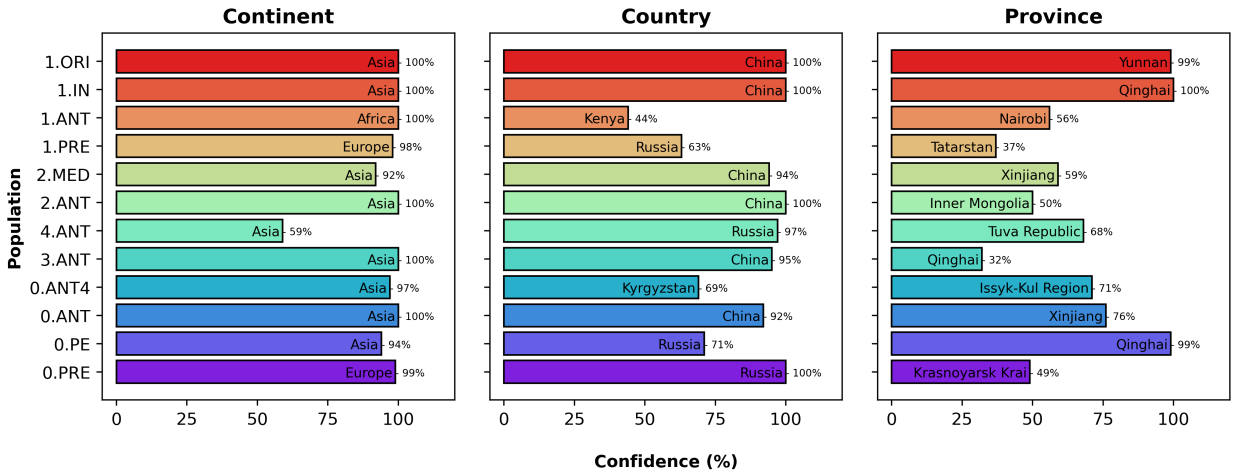

Supplement: Supplementary file 2 — Supplementary Figures [file 42003_2022_4394_MOESM2_ESM.pdf]
